# Supplementary material for: Manual Muscle Testing—Force Profiles and Their Reproducibility
Source: Diagnostics (Basel). 2020 Nov 25;10(12):996. doi: 10.3390/diagnostics10120996 (PMC7759939; doi:10.3390/diagnostics10120996)
Supplement: Supplementary file 1 [file diagnostics-10-00996-s001.pdf]

diagnostics

Supplementary material

Manual Muscle Testing – Force Profiles and their Reproducibility

Content

Table S1: Starting force (F_{start}) 2

Table S2: Maximum force (F_{max}) 3

Table S3: Slope from start to F_{max} (slope_2max) 4

Table S4: 1st derivative of force curves 5

Table S5: Slope in the linear section XYZ (slope_XYZ) 6

Table S6: Intraclass correlation coefficient & normalized mean Euclidean distance (MED) 8

Table S1: Starting force (F_{start})

Table S1. Starting force (F_{start} (N)) of each tester and trial sorted by groups experienced (Exp), little experienced (LitExp) and beginners (Beg). The arithmetic means (M), standard deviations (SD) and coefficients of variation (CV) are given per tester.

	tester	M1	M2	M3	M4	M5	M6	M7	M8	M9	M10	M	SD	CV
Experienced	Exp_1	6.34	7.84	4.19	6.19	3.19	3.44	3.55	4.72	5.30	5.23	5.00	1.49	0.30
	Exp_2	23.41	10.49	19.92	24.66	10.17	18.71	14.95	17.81	16.84	13.03	17.00	4.95	0.29
	Exp_3	8.64	9.08	16.58	12.84	13.47	15.03	11.88	17.76	12.94	19.04	13.73	3.44	0.25
	Exp_4	8.52	10.10	17.43	15.32	0.69	17.15	17.99	15.59	13.62	12.09	12.85	5.32	0.41
	Exp_5	10.71	13.60	6.42	7.55	11.23	11.44	12.21	10.58	1.25	11.81	9.68	3.65	0.38
	Exp_6	5.82	5.55	9.71	4.61	8.71	10.84	8.15	8.40	9.20	8.73	7.97	2.00	0.25
	Exp_7	16.13	27.38	20.66	27.45	24.26	21.35	19.09	18.65	15.90	9.41	20.03	5.54	0.28
	Exp_8	22.07	27.13	26.76	21.68	11.51	20.15	22.16	29.48	20.03	20.20	22.12	5.01	0.23
	Exp_9	12.55	11.38	6.66	11.40	8.95	8.20	10.30	10.47	9.66	12.24	10.18	1.86	0.18
Little experienced	LitExp_1	18.16	9.75	14.50	14.79	7.34	15.15	10.45	11.44	12.37	13.61	12.75	3.13	0.25
	LitExp_2	7.59	25.61	19.22	16.17	25.44	30.57	27.85	25.01	25.96	28.52	23.19	6.95	0.30
	LitExp_3	8.82	7.04	8.93	8.01	9.24	7.34	7.30	8.06	8.59	5.32	7.86	1.17	0.15
	LitExp_4	7.36	7.34	8.80	10.40	9.94	11.38	10.66	6.50	7.81	9.74	8.99	1.67	0.19
	LitExp_5	20.23	19.77	20.15	18.43	21.15	26.27	19.01	31.21	16.83	23.35	21.64	4.27	0.20
	LitExp_6	4.17	4.13	4.67	2.19	3.43	3.75	3.30	4.15	4.27	4.92	3.90	0.78	0.20
	LitExp_7	7.16	7.27	9.52	6.48	2.70	8.21	10.57	8.28	6.04	5.68	7.19	2.19	0.31
	LitExp_8	7.88	8.35	12.15	8.40	10.55	5.83	3.53	7.15	5.83	2.41	7.21	2.97	0.41
Beginners	Beg_1	1.73	2.61	5.01	4.16	3.62	1.80	4.37	3.84	2.65	3.50	3.33	1.10	0.33
	Beg_2	3.49	2.41	2.55	2.90	2.56	3.57	1.98	1.70	0.58	0.96	2.27	0.99	0.43
	Beg_3	9.58	10.66	10.01	12.50	5.94	10.51	3.67	7.82	8.32	8.03	8.70	2.55	0.29
	Beg_4	7.79	9.26	7.91	6.63	9.41	10.44	8.32	8.65	7.66	9.23	8.53	1.10	0.13
	Beg_5	11.68	3.73	6.26	5.89	10.50	7.06	6.77	6.08	4.95	3.95	6.69	2.58	0.39
	Beg_6	7.40	0.80	1.43	1.31	0.93	1.72	0.87	0.72	0.81	0.34	1.63	2.06	1.27
	Beg_7	12.02	12.36	7.25	4.59	7.49	7.14	9.74	7.25	5.20	5.80	7.88	2.68	0.34
	Beg_8	8.71	11.92	15.66	10.79	12.74	0.83	3.88	1.50	1.57	6.17	7.38	5.34	0.72
	Beg_9	12.22	11.45	6.40	8.30	9.20	7.95	6.97	8.02	11.66	4.75	8.69	2.45	0.28
	Beg_10	1.09	0.79	3.80	1.00	5.51	7.28	6.24	0.38	4.84	9.28	4.02	3.12	0.78
	Beg_11	8.46	7.50	10.03	4.25	12.13	9.84	7.48	12.49	14.10	5.40	9.17	3.16	0.34
	Beg_12	0.92	-1.23	1.08	1.08	1.34	-1.29	0.93	1.57	1.35	-1.55	0.42	1.25	2.96

Table S2: Maximum force (F_{max})

Table S2. Maximum force (F_{max} (N)) of each tester and trial sorted by groups experienced (Exp), little experienced (LitExp) and beginners (Beg). The arithmetic means (M), standard deviations (SD) and coefficients of variation (CV) are given per tester.

	tester	M1	M2	M3	M4	M5	M6	M7	M8	M9	M10	M	SD	CV
Experienced	Exp_1	25.56	28.82	27.53	29.97	30.48	29.59	27.61	30.79	28.26	28.10	28.67	1.60	0.06
	Exp_2	27.82	27.31	29.93	29.11	31.71	30.96	30.11	29.24	30.23	30.40	29.68	1.35	0.05
	Exp_3	23.91	27.84	26.52	29.27	26.97	25.90	26.25	23.14	24.53	24.97	25.93	1.86	0.07
	Exp_4	20.53	17.56	23.44	27.21	25.01	26.15	25.90	24.23	25.16	24.98	24.02	2.90	0.12
	Exp_5	15.17	13.71	14.30	13.08	13.54	13.50	13.29	14.04	14.70	16.11	14.14	0.95	0.07
	Exp_6	31.81	34.61	32.77	34.04	32.51	35.74	33.43	33.89	34.85	37.60	34.13	1.69	0.05
	Exp_7	18.27	21.17	19.83	20.05	18.84	17.44	16.35	17.89	16.46	17.96	18.43	1.56	0.08
	Exp_8	21.64	19.68	18.53	19.55	22.64	18.09	18.70	21.27	18.38	21.64	20.01	1.65	0.08
	Exp_9	15.98	12.65	12.31	14.66	14.95	13.18	12.98	12.88	13.32	11.09	13.40	1.42	0.11
Little experienced	LitExp_1	16.00	17.67	14.83	14.08	14.54	11.85	12.43	13.22	11.62	11.77	13.80	2.01	0.15
	LitExp_2	40.32	39.01	35.19	33.78	37.41	33.99	37.45	35.48	38.46	37.50	36.86	2.18	0.06
	LitExp_3	8.36	9.82	9.63	9.54	9.37	9.36	9.33	10.02	10.01	8.04	9.35	0.66	0.07
	LitExp_4	15.40	14.88	15.07	14.16	13.51	15.10	16.66	15.67	16.88	16.40	15.37	1.07	0.07
	LitExp_5	15.26	15.16	15.84	15.09	14.60	15.67	16.10	15.77	15.02	14.34	15.29	0.56	0.04
	LitExp_6	13.66	15.26	15.07	14.10	12.52	13.77	15.44	14.23	12.89	13.38	14.03	0.99	0.07
	LitExp_7	9.19	8.50	6.99	6.00	6.50	6.86	6.83	6.67	6.46	5.46	6.95	1.11	0.16
	LitExp_8	14.49	13.70	14.23	13.23	11.62	12.57	12.79	12.40	13.09	14.21	13.23	0.93	0.07
Beginners	Beg_1	10.89	14.42	14.09	11.96	14.24	12.38	12.06	10.91	10.90	10.06	12.19	1.58	0.13
	Beg_2	27.79	32.44	30.50	33.47	33.45	35.20	33.14	35.44	32.04	36.89	33.04	2.60	0.08
	Beg_3	16.26	14.57	14.03	14.22	14.70	13.70	15.08	17.50	16.01	15.79	15.19	1.19	0.08
	Beg_4	21.47	20.28	18.14	19.87	18.89	22.40	22.80	21.43	20.09	21.60	20.70	1.50	0.07
	Beg_5	13.34	13.41	12.85	13.57	12.94	13.19	13.44	13.35	14.03	12.39	13.25	0.45	0.03
	Beg_6	23.21	28.57	26.99	28.44	28.35	27.86	28.75	29.01	28.10	28.29	27.76	1.69	0.06
	Beg_7	11.53	11.69	10.61	10.43	10.43	15.04	12.97	14.47	12.20	12.44	12.18	1.61	0.13
	Beg_8	15.36	18.04	16.83	17.02	17.96	18.35	18.32	16.53	15.04	17.70	17.11	1.19	0.07
	Beg_9	8.01	9.10	9.24	9.88	10.64	9.60	10.20	10.45	9.47	10.50	9.71	0.81	0.08
	Beg_10	19.51	21.19	21.23	22.44	25.78	24.16	25.33	23.77	22.61	21.52	22.76	1.99	0.09
	Beg_11	18.20	19.37	18.84	18.40	18.73	17.98	18.26	18.67	16.99	17.82	18.33	0.65	0.04
	Beg_12	13.36	11.90	11.87	11.30	11.96	11.09	11.97	12.76	13.27	12.30	12.18	0.76	0.06

Table S3: Slope from start to F_{max} (slope_2max)

Table S3. Slope from 2s to F_{max} (N/s) of each tester and trial sorted by groups experienced (Exp), little experienced (LitExp) and beginners (Beg). The arithmetic means (M), standard deviations (SD) and coefficients of variation (CV) are given per tester.

	tester	M1	M2	M3	M4	M5	M6	M7	M8	M9	M10	M	SD	CV
Experienced	Exp_1	78.36	80.36	68.12	75.56	80.93	77.13	74.71	78.24	85.60	76.91	77.59	4.56	0.06
	Exp_2	69.76	56.13	64.42	66.93	75.77	68.85	72.18	58.69	71.44	63.68	66.79	6.14	0.09
	Exp_3	75.98	83.24	85.41	92.99	83.68	81.84	82.16	70.27	79.03	82.48	81.71	5.95	0.07
	Exp_4	67.74	58.69	71.33	82.76	76.29	83.59	78.64	69.69	77.56	81.79	74.81	7.90	0.11
	Exp_5	50.22	46.80	52.90	45.18	41.68	47.08	48.44	50.23	49.49	53.93	48.59	3.62	0.07
	Exp_6	118.4	119.7	122.6	118.4	120.3	124.8	116.0	119.3	128.5	129.1	121.7	4.45	0.04
	Exp_7	48.35	71.41	69.51	77.77	72.91	67.70	65.54	70.86	64.52	71.72	68.03	7.89	0.12
	Exp_8	93.69	85.68	81.67	84.71	95.53	81.44	80.33	89.64	82.05	94.43	86.92	5.91	0.07
	Exp_9	25.71	20.91	21.01	24.97	23.41	24.10	21.01	19.85	25.99	14.59	22.15	3.45	0.16
Little experienced	LitExp_1	48.94	62.13	43.26	46.21	48.77	39.38	34.94	43.09	38.41	38.75	44.39	7.77	0.18
	LitExp_2	94.29	91.71	79.99	62.74	70.26	67.54	75.32	64.54	80.03	78.17	76.46	10.69	0.14
	LitExp_3	19.04	19.01	17.45	17.24	16.08	24.24	20.32	26.37	17.57	14.32	19.16	3.67	0.19
	LitExp_4	41.97	43.37	43.07	42.39	38.01	43.23	43.80	40.41	45.50	44.73	42.65	2.16	0.05
	LitExp_5	37.08	39.29	41.79	38.99	33.44	37.65	43.68	35.51	38.74	34.33	38.05	3.18	0.08
	LitExp_6	30.13	40.68	37.54	36.13	31.44	37.50	39.27	36.69	33.42	37.11	35.99	3.35	0.09
	LitExp_7	22.16	18.97	18.80	13.93	13.86	14.85	15.36	14.92	15.97	15.45	16.43	2.68	0.16
	LitExp_8	30.45	30.75	26.34	27.99	24.66	31.32	34.50	30.14	30.23	35.73	30.21	3.35	0.11
Beginners	Beg_1	12.75	23.39	24.71	19.46	20.54	23.18	15.73	21.96	19.55	11.72	19.30	4.51	0.23
	Beg_2	75.79	67.91	63.18	70.70	69.88	85.67	77.77	65.63	82.25	94.11	75.29	9.79	0.13
	Beg_3	39.48	34.24	37.53	36.19	40.48	34.24	30.74	37.20	54.26	52.45	39.68	7.73	0.19
	Beg_4	64.77	63.67	61.18	63.29	62.68	69.51	71.36	64.41	64.11	69.56	65.45	3.42	0.05
	Beg_5	32.70	25.73	26.47	25.38	24.49	27.31	26.72	29.76	33.66	29.77	28.20	3.14	0.11
	Beg_6	75.13	82.94	71.86	84.59	80.71	76.86	79.47	88.30	78.41	83.50	80.18	4.87	0.06
	Beg_7	26.93	26.63	24.05	25.07	25.36	36.17	28.98	32.84	25.76	28.38	28.02	3.81	0.14
	Beg_8	48.28	61.24	52.22	48.51	54.49	54.92	52.66	46.58	46.35	54.17	51.94	4.63	0.09
	Beg_9	12.13	16.24	15.74	17.20	19.09	13.79	21.60	16.49	26.70	17.08	17.61	4.12	0.23
	Beg_10	27.36	35.73	48.06	47.71	55.57	53.93	50.83	50.72	47.44	47.11	46.45	8.57	0.18
	Beg_11	55.61	57.58	62.77	64.13	57.47	53.44	53.74	57.87	53.28	58.25	57.41	3.72	0.06
	Beg_12	34.69	37.08	29.28	27.12	29.61	26.49	30.49	30.12	34.94	32.50	31.23	3.48	0.11

Table S4: 1st derivative of force curves

Table S4. Averaged values of 1st derivative of the force profile from 0.5s to F_{max} (N/s) of each tester and trial sorted by groups experienced (Exp), little experienced (LitExp) and beginners (Beg). The arithmetic means (M), standard deviations (SD) and coefficients of variation (CV) are given per tester.

	**tester**	**M1**	**M2**	**M3**	**M4**	**M5**	**M6**	**M7**	**M8**	**M9**	**M10**	**M**	**SD**	**CV**
Experienced	Exp_1	61.59	62.61	58.36	66.02	67.39	64.49	59.59	74.91	76.16	72.80	66.39	6.34	0.10
	Exp_2	63.66	45.38	53.61	56.38	55.58	63.60	50.05	46.22	59.06	49.66	54.32	6.56	0.12
	Exp_3	51.97	48.91	59.04	72.05	81.14	74.26	72.42	85.84	58.41	81.56	68.56	13.08	0.19
	Exp_4	66.23	59.77	72.51	78.25	79.47	75.96	79.29	73.10	73.63	76.80	73.50	6.25	0.08
	Exp_5	54.15	54.32	59.52	57.91	55.42	55.88	59.62	62.82	60.01	67.40	58.71	4.15	0.07
	Exp_6	101.4	101.5	109.4	107.8	104.6	127.9	100.4	112.0	109.6	119.3	109.4	8.70	0.08
	Exp_7	48.47	81.59	109.0	106.1	83.00	93.47	85.36	97.73	87.87	103.1	89.58	17.43	0.19
	Exp_8	138.4	105.7	88.81	96.29	123.4	127.5	106.6	126.0	125.8	119.8	115.8	15.71	0.14
	Exp_9	21.36	17.21	18.70	21.36	20.92	19.01	17.23	17.37	21.12	13.76	18.80	2.48	0.13
Little experienced	LitExp_1	44.16	68.02	52.70	49.43	36.80	42.01	39.38	41.01	38.81	39.32	45.16	9.45	0.21
	LitExp_2	74.75	74.91	61.34	48.36	56.34	49.36	55.69	57.20	68.72	67.59	61.42	9.68	0.16
	LitExp_3	19.31	19.03	18.13	17.57	16.70	24.46	20.31	26.69	17.96	15.46	19.56	3.49	0.18
	LitExp_4	43.84	46.46	44.26	41.51	31.49	45.82	53.12	44.28	48.94	46.72	44.64	5.61	0.13
	LitExp_5	38.91	37.88	35.14	34.42	28.35	32.41	36.49	33.95	35.06	28.96	34.16	3.46	0.10
	LitExp_6	32.84	33.99	34.02	34.22	31.57	37.17	35.32	37.49	30.70	36.58	34.39	2.29	0.07
	LitExp_7	25.32	20.99	15.38	14.66	14.60	15.33	15.37	15.62	17.49	15.41	17.02	3.48	0.20
	LitExp_8	26.52	28.81	28.06	29.49	23.84	27.51	32.97	29.52	26.26	39.20	29.22	4.26	0.15
Beginners	Beg_1	12.41	20.35	23.20	15.03	18.15	20.53	14.66	18.32	17.36	10.10	17.01	3.99	0.23
	Beg_2	66.91	76.28	74.26	68.48	64.17	67.46	73.16	55.64	62.20	77.36	68.59	6.84	0.10
	Beg_3	34.33	28.35	30.66	31.60	27.32	37.04	30.82	48.13	42.18	35.63	34.61	6.48	0.19
	Beg_4	68.91	61.18	52.86	59.34	59.82	77.37	77.50	62.28	59.04	73.85	65.21	8.60	0.13
	Beg_5	28.53	19.57	20.04	20.34	19.80	23.04	19.81	24.09	29.12	24.44	22.88	3.64	0.16
	Beg_6	51.84	95.13	67.43	93.33	90.44	84.74	92.73	93.96	80.45	72.55	82.26	14.34	0.17
	Beg_7	22.90	23.87	22.31	20.45	22.67	31.08	24.58	28.58	23.22	25.03	24.47	3.15	0.13
	Beg_8	57.05	61.88	54.90	60.25	55.04	59.49	54.80	55.74	40.09	52.48	55.17	6.04	0.11
	Beg_9	13.97	15.24	15.84	17.37	16.80	16.66	21.53	17.57	22.33	17.18	17.45	2.61	0.15
	Beg_10	24.42	31.31	38.99	40.82	49.96	49.94	48.87	43.65	47.74	41.66	41.74	8.45	0.20
	Beg_11	50.96	48.38	57.34	52.85	59.31	65.21	52.81	48.92	53.34	60.60	54.97	5.47	0.10
	Beg_12	38.00	45.30	32.48	26.41	32.63	28.91	35.92	34.02	39.16	41.08	35.39	5.70	0.16

Table S5: Slope in the linear section XYZ (slope_XYZ)

Table S5. Slope in the linear section XY, YZ, XZ and the difference of YZ and XY (N/s) of each tester and trial sorted by groups experienced (Exp), little experienced (LitExp) and beginners (Beg). The arithmetic means (M), standard deviations (SD) and coefficients of variation (CV) are given per tester.

	Tester	slope	M1	M2	M3	M4	M5	M6	M7	M8	M9	M10	M	SD	CV
Experienced	Exp_1	XY	104.47	97.48	177.65	187.29	161.60	126.77	171.44	148.04	174.34	134.46	148.36	31.50	0.21
		YZ	146.63	113.07	149.19	151.57	175.86	153.60	194.88	123.78	123.20	217.04	154.88	32.89	0.21
		XZ	122.01	104.70	162.18	167.55	168.43	138.90	182.41	134.83	144.38	166.05	149.14	24.31	0.16
	Exp_2	XY	94.10	92.37	112.48	116.55	100.34	116.81	123.58	122.57	66.95	112.53	105.83	17.58	0.17
		YZ	119.69	118.00	116.96	106.94	123.92	133.79	136.11	140.59	101.93	122.72	122.07	12.31	0.10
		XZ	105.36	103.62	114.68	111.54	110.89	124.73	129.54	130.96	80.82	117.40	112.95	14.70	0.13
	Exp_3	XY	106.64	188.38	240.88	208.09	224.26	174.05	247.63	153.40	182.27	226.78	195.24	43.58	0.22
		YZ	161.79	155.20	218.62	222.61	213.41	151.26	183.96	186.09	170.64	205.81	186.94	26.92	0.14
		XZ	128.55	170.19	229.21	215.10	218.70	161.86	211.10	168.17	176.27	215.79	189.49	32.87	0.17
	Exp_4	XY	159.83	135.65	151.27	153.40	185.90	192.87	154.95	171.00	150.51	197.64	165.30	20.65	0.12
		YZ	128.27	127.61	144.61	118.11	113.61	183.22	171.70	163.92	125.29	214.98	149.13	33.23	0.22
		XZ	142.33	131.50	147.87	133.46	141.03	187.92	162.90	167.38	136.75	205.94	155.71	25.05	0.16
	Exp_5	XY	206.63	220.55	237.74	188.63	201.24	210.27	146.49	275.45	140.01	197.53	202.46	39.76	0.20
		YZ	193.21	197.85	219.17	166.59	228.99	270.35	181.08	275.45	171.68	188.12	209.25	38.68	0.18
		XZ	199.69	208.59	228.08	176.92	214.22	236.56	161.96	275.45	154.24	192.71	204.84	36.49	0.18
	Exp_6	XY	339.18	204.56	261.37	292.95	253.13	217.79	241.14	237.49	231.00	302.35	258.10	41.79	0.16
		YZ	350.61	237.46	334.89	283.02	292.61	280.52	204.97	223.14	310.79	396.64	291.46	59.70	0.20
		XZ	344.80	219.79	293.60	287.90	271.44	245.21	221.59	230.09	265.02	343.14	272.26	45.79	0.17
	Exp_7	XY	86.58	156.12	216.10	226.02	171.09	206.17	155.76	179.09	192.23	198.02	178.72	40.18	0.22
		YZ	71.98	114.08	209.13	393.28	177.67	201.32	182.31	201.73	173.63	228.88	195.40	83.90	0.43
		XZ	78.61	131.83	212.56	287.07	174.32	203.72	167.99	189.73	182.46	212.33	184.06	54.61	0.30
	Exp_8	XY	255.79	327.24	279.70	262.72	308.45	432.76	295.85	386.43	500.78	530.82	358.05	99.76	0.28
		YZ	393.16	332.88	386.82	314.40	358.20	657.16	366.85	293.90	462.26	530.82	409.65	111.7	0.27
		XZ	309.93	330.04	324.65	286.24	331.47	521.86	327.55	333.88	480.75	530.82	377.72	93.96	0.25
	Exp_9	XY	14.24	13.01	43.91	19.44	13.90	21.77	20.54	17.97	15.71	18.51	19.90	8.94	0.45
		YZ	23.61	20.48	16.84	28.04	19.53	24.40	15.80	23.66	24.48	17.09	21.39	4.05	0.19
		XZ	17.77	15.91	24.34	22.96	16.24	23.01	17.86	20.43	19.14	17.77	19.54	3.00	0.15
Little experienced	LitExp_1	XY	110.55	143.25	127.61	123.30	162.12	115.14	112.89	101.30	118.72	116.61	123.15	17.67	0.14
		YZ	123.61	114.03	154.76	132.79	153.40	130.66	133.98	115.78	146.11	153.93	135.90	15.51	0.11
		XZ	116.71	126.98	139.88	127.87	157.64	122.41	122.53	108.06	131.00	132.70	128.58	13.48	0.10
	LitExp_2	XY	155.71	103.42	103.97	127.94	98.38	108.25	118.14	134.90	127.48	100.52	117.87	18.53	0.16
		YZ	187.44	118.47	127.37	71.26	108.57	71.09	86.25	86.15	135.25	163.50	115.53	39.04	0.34
		XZ	170.11	110.44	114.49	91.54	103.22	85.82	99.71	105.15	131.25	124.50	113.62	24.19	0.21
	LitExp_3	XY	80.42	112.07	113.76	126.48	122.55	133.00	134.63	161.09	100.19	121.29	120.55	21.55	0.18
		YZ	80.42	88.42	85.84	114.14	153.18	100.85	85.56	85.45	85.38	99.80	97.90	21.95	0.22
		XZ	80.42	98.85	97.85	119.99	136.16	114.72	104.63	111.66	92.19	109.50	106.60	15.59	0.15
	LitExp_4	XY	71.61	70.87	36.86	70.52	70.13	146.65	151.32	114.68	81.95	134.07	94.87	38.95	0.41
		YZ	103.49	73.00	90.67	106.04	89.55	118.49	126.69	229.37	68.41	98.10	110.38	45.57	0.41
		XZ	84.65	71.92	52.41	84.71	78.66	131.08	137.91	152.91	74.57	113.30	98.21	33.29	0.34
	LitExp_5	XY	76.78	70.14	59.76	57.37	31.75	57.59	69.29	59.52	77.17	33.42	59.28	15.91	0.27
		YZ	95.97	71.49	73.99	62.72	27.54	55.71	107.48	48.66	77.98	42.37	66.39	24.29	0.37
		XZ	85.31	70.81	66.12	59.92	29.49	56.63	84.26	53.54	77.57	37.37	62.10	18.69	0.30
	LitExp_6	XY	73.23	61.87	71.08	79.49	67.46	105.53	105.17	112.55	92.26	91.16	85.98	17.87	0.21
		YZ	67.35	65.67	86.97	104.78	59.89	84.96	109.74	85.62	92.26	68.37	82.56	16.97	0.21
		XZ	70.17	63.72	78.22	90.39	63.45	94.13	107.41	97.26	92.26	78.14	83.52	15.00	0.18
	LitExp_7	XY	43.76	24.97	31.01	46.36	25.72	37.39	29.90	28.33	45.24	34.58	34.73	8.09	0.23
		YZ	43.13	35.19	36.85	26.76	21.55	40.30	36.20	36.56	47.26	25.52	34.93	8.10	0.23
		XZ	43.45	29.21	33.68	33.93	23.45	38.79	32.75	31.92	46.23	29.37	34.28	6.85	0.20
	LitExp_8	XY	51.14	64.00	64.31	98.35	47.88	88.06	60.89	87.50	47.91	120.19	73.02	24.38	0.33
		YZ	32.46	67.53	67.10	63.95	50.65	86.82	70.07	71.54	66.53	73.77	65.04	14.53	0.22
		XZ	39.71	65.72	65.67	77.51	49.23	87.44	65.16	78.72	55.70	91.42	67.63	16.51	0.24

		XY	26.50	58.70	37.37	51.69	16.36	18.38	19.47	25.86	14.69	24.56	29.36	15.17	0.52
	Beg_1	YZ	11.92	57.75	49.38	25.40	30.30	9.97	7.92	19.75	27.57	7.22	24.72	17.43	0.70
		XZ	16.45	58.22	42.54	34.06	21.25	12.93	11.26	22.40	19.17	11.16	24.94	15.45	0.62
		XY	146.59	232.32	415.55	192.04	106.88	78.47	119.53	146.69	53.45	81.14	157.27	105.7	0.67
	Beg_2	YZ	75.32	57.24	339.99	218.92	97.94	200.75	70.83	69.81	198.92	152.70	148.24	91.52	0.62
		XZ	99.51	91.85	373.99	204.60	102.21	112.84	88.96	94.60	84.26	105.97	135.88	90.56	0.67
		XY	142.44	88.20	17.25	52.43	28.22	81.95	68.48	553.93	119.92	67.63	122.05	156.4	1.28
	Beg_3	YZ	212.72	22.79	103.49	129.14	111.80	19.23	26.04	113.72	161.95	137.05	103.79	64.05	0.62
		XZ	170.63	36.22	29.57	74.58	45.07	31.15	37.73	188.70	137.80	90.56	84.20	60.73	0.72
		XY	129.98	211.69	141.22	151.12	247.07	183.10	219.26	142.06	150.45	190.90	176.69	39.74	0.22
	Beg_4	YZ	186.35	218.67	121.87	172.52	183.47	209.26	209.02	168.20	169.90	124.65	176.39	33.09	0.19
		XZ	153.14	215.12	130.83	161.12	210.57	195.31	214.02	154.03	159.58	150.82	174.45	31.07	0.18
		XY	36.24	28.54	40.39	43.36	26.49	58.28	30.95	49.62	28.14	29.21	37.12	10.66	0.29
	Beg_5	YZ	56.15	48.56	79.76	59.97	37.88	31.48	28.98	70.82	38.44	62.97	51.50	17.22	0.33
		XZ	44.05	35.95	53.63	50.33	31.18	40.88	29.93	58.36	32.50	39.91	41.67	9.83	0.24
		XY	167.39	162.01	372.96	306.59	171.69	267.93	316.85	209.24	172.31	201.11	234.81	75.71	0.32
	Beg_6	YZ	206.95	201.64	187.80	214.61	164.58	162.67	233.06	213.96	188.83	142.32	191.64	28.09	0.15
		XZ	185.08	179.67	249.81	252.48	168.06	202.44	268.57	211.57	180.19	166.69	206.46	37.74	0.18
Beginners		XY	35.01	40.25	30.07	28.04	45.27	43.92	29.32	46.08	30.15	44.37	37.25	7.46	0.20
	Beg_7	YZ	34.90	44.29	25.01	29.07	32.27	42.77	22.56	31.61	25.20	38.49	32.62	7.50	0.23
		XZ	34.95	42.17	27.31	28.55	37.68	43.34	25.50	37.50	27.45	41.22	34.57	6.83	0.20
		XY	149.21	108.59	100.04	163.65	124.95	132.34	47.06	108.12	97.68	100.36	113.20	32.23	0.28
	Beg_8	YZ	81.02	133.08	143.54	96.49	160.17	55.90	58.36	69.61	74.12	100.36	97.26	36.79	0.38
		XZ	105.02	119.59	117.91	121.40	140.39	78.59	52.10	84.69	84.28	100.36	100.43	25.93	0.26
		XY	35.57	44.40	50.37	35.91	51.65	46.38	54.09	32.45	77.41	31.04	45.93	13.84	0.30
	Beg_9	YZ	40.73	24.25	30.53	27.86	41.08	33.27	52.94	55.74	152.29	88.07	54.68	39.03	0.71
		XZ	37.97	31.37	38.01	31.38	45.76	38.75	53.51	41.02	102.65	45.90	46.63	20.80	0.45
		XY	31.89	36.79	61.27	46.26	95.07	68.71	103.99	61.69	96.87	40.37	64.29	26.51	0.41
	Beg_10	YZ	25.75	49.15	58.19	63.82	60.07	55.00	45.10	50.37	52.82	80.90	54.12	14.10	0.26
		XZ	28.50	42.08	59.69	53.64	73.62	61.09	62.92	55.46	68.36	53.87	55.92	12.96	0.23
		XY	100.31	124.16	117.70	109.39	113.45	84.01	98.94	90.21	90.09	127.59	105.59	15.15	0.14
	Beg_11	YZ	85.43	111.75	104.40	121.95	167.08	112.36	94.25	128.96	148.82	112.78	118.78	24.49	0.21
		XZ	92.28	117.63	110.65	115.33	135.14	96.14	96.54	106.16	112.24	119.73	110.18	13.00	0.12
		XY	92.94	128.30	59.73	62.25	83.83	60.13	81.53	64.18	77.48	71.80	78.22	20.90	0.27
	Beg_12	YZ	67.55	104.25	71.90	56.25	74.75	56.10	79.86	55.63	77.95	90.01	73.43	15.77	0.21
		XZ	78.24	115.03	65.26	59.10	79.03	58.04	80.69	59.60	77.71	79.88	75.26	16.88	0.22

Table S6: Intraclass correlation coefficient & normalized mean Euclidean distance (MED)

Table S6. Values of Intraclass correlation coefficient (ICC(3,1)) and normalized mean Euclidean distance (MED (%)) calculated by using the 10 force trials of each tester sorted by groups experienced (Exp), little experienced (LitExp) and beginners (Beg).

	tester	ICC(3,1)	MED [%]
Experienced	Exp_1	0.992	25.754
	Exp_2	0.995	18.412
	Exp_3	0.991	25.898
	Exp_4	0.986	30.211
	Exp_5	0.994	20.769
	Exp_6	0.995	18.205
	Exp_7	0.979	35.571
	Exp_8	0.989	18.770
	Exp_9	0.931	44.888
Little experienced	LitExp_1	0.975	35.566
	LitExp_2	0.983	29.176
	LitExp_3	0.981	46.026
	LitExp_4	0.983	37.798
	LitExp_5	0.976	42.694
	LitExp_6	0.983	37.170
	LitExp_7	0.948	57.104
	LitExp_8	0.982	40.215
Beginners	Beg_1	0.834	77.501
	Beg_2	0.931	66.159
	Beg_3	0.992	25.321
	Beg_4	0.983	40.873
	Beg_5	0.947	64.978
	Beg_6	0.913	78.768
	Beg_7	0.993	22.837
	Beg_8	0.979	39.300
	Beg_9	0.988	31.341
	Beg_10	0.976	33.660
	Beg_11	0.983	35.890
	Beg_12	0.950	53.543
